# Supplementary material for: UMOD Polymorphisms Associated with Kidney Function, Serum Uromodulin and Risk of Mortality among Patients with Chronic Kidney Disease, Results from the C-STRIDE Study
Source: Genes (Basel). 2021 Oct 23;12(11):1687. doi: 10.3390/genes12111687 (PMC8620616; doi:10.3390/genes12111687)
Supplement: Supplementary file 1 [file genes-12-01687-s001.zip › genes-1416218-supplementary.pdf]

**Table of Contents for Supplemental Materials**

**Table S1.** Measurements of linkage disequilibrium between the studied polymorphisms;

**Table S2.** Characteristics of study participants stratified by genotypes of rs13333226/rs4293393;

**Table S3.** Characteristics of study participants stratified by genotypes of rs6497476;

**Table S4.** Characteristics of study participants between included and excluded participants;

**Table S5.** Genetic association between genotypes of rs4293393 or rs6497476 and eGFR or uromodulin;

**Table S6.** Genetic association between genotypes of rs11864909 or haplotypes composed of rs11864909, rs4293393 and rs6497476 and eGFR or uromodulin, by age, hypertension status, eGFR, ACR and etiology of CKD;

**Table S7.** Incidence rates for all-cause mortality, by genotypes of rs4293393 and rs6497476 and 24h urinary sodium excretion;

**Table S8.** eGFR slope stratified by genotypes of rs11864909, rs4293393 and rs6497476 and haplotypes involving the variants

**Table S9.** eGFR slope stratified by genotypes of rs11864909, rs4293393 and rs6497476 and haplotypes composed of the variants among the participants with ≥3 times of measures of eGFR;

**Figure S1.** The distribution of 24-hour urinary sodium excretion through genotypes of rs4293393 and rs6497476. A. 24-hour urinary sodium excretion through genotypes of rs4293393; B. 24-hour urinary sodium excretion through genotypes of rs6497476.

**Table S1.** Measurements of linkage disequilibrium between the studied polymorphisms

| | rs11864909 | rs4293393 | rs6497476 | rs13333226 |
|---|---|---|---|---|
| rs11864909 | - | D': 0.577; LOD: 3.22; r-squared: 0.005 | D': 1.0; LOD: 9.54; r-squared: 0.012 | D': 0.577; LOD: 3.22; r-squared: 0.005 |
| rs4293393 | - | - | D': 1.0; LOD: 416.67; r-squared: 0.864 | D': 1.0; LOD: 531.83; r-squared: 1.0 |
| rs6497476 | - | - | - | D': 1.0; LOD: 416.67; r-squared: 0.864 |
| rs13333226 | - | - | - | - |

Abbreviation: LOD, logarithm of the odds score.

**Table S2.** Characteristics of study participants stratified by genotypes of rs13333226/rs4293393

| Characteristics | A/A n=2333 | G/A & G/G n=398 | *P*-value |
|---|---|---|---|
| Age, years | 48.99±13.80 | 48.62±13.84 | 0.62 |
| Male, n(%) | 1405(60.22%) | 229(57.54%) | 0.31 |
| High school and above, n(%) | 1277(55.26%) | 232(58.29%) | 0.26 |
| Current and ever smoking, n(%) | 884(39.12%) | 159(41.09%) | 0.46 |
| Body mass index, kg/m2 | 24.51±3.65 | 24.40±3.44 | 0.59 |
| Systolic blood pressure, mmHg | 129.85±18.18 | 127.97±16.66 | 0.07 |
| Diastolic blood pressure, mmHg | 81.02±10.97 | 80.64±10.80 | 0.55 |
| Using anti-hypertensive medication, n(%) | 1400(73.61%) | 220(69.40%) | 0.12 |
| Diabetes mellitus, n(%) | 534(25.43%) | 91(26.07%) | 0.80 |
| History of CVD, n(%) | 235(10.07%) | 42(10.55%) | 0.77 |
| Creatinine, μmol/L | 143(100, 205) | 140.55(100, 208) | 0.71 |
| eGFR, ml/min/1.73m2 | 51.49±30.43 | 51.85±30.55 | 0.83 |
| eGFR<60ml/min/1.73m2, n(%) | 1589(68.11%) | 269(67.59%) | 0.84 |
| ACR, mg/g | 427.91(111.10, 985.52) | 484.42(131.70, 1003.49) | 0.26 |
| Albuminuria groups, n(%) | | | 0.06 |
| <30 mg/g | 281(12.31%) | 34(8.83%) | |
| 30-299 mg/g | 644(28.21%) | 126(32.73%) | |
| ≥300 mg/g | 1358(59.48%) | 225(58.44%) | |
| 24h urinary sodium excretion (mmol/24h) | 146.39±80.47 | 145.02±80.81 | 0.76 |
| Uromodulin, ng/mL | 91.42±61.23 | 92.65±62.25 | 0.73 |
| Etiology of CKD | | | 0.41 |
| Diabetic nephropathy | 341(15.04%) | 51(13.21%) | |
| Glomerulonephritis | 1360(59.96%) | 245(63.47%) | |
| Others | 567(25.00%) | 90(23.32%) | |

Note: Number of missing: education-22, smoking status-84, body mass index-263, SBP-379, DBP-379, using anti-hypertensive medication-512, diabetes mellitus-282, etiology of CKD-77, ACR-63, uromodulin-327.

Abbreviation: CVD, cardiovascular disease; eGFR, estimated glomerular filtration rate; ACR, albumin creatinine ratio ; CKD, chronic kidney disease.

**Table S3.** Characteristics of study participants stratified by genotypes of rs6497476

| Characteristics | T/T n=2382 | T/C & C/C n=349 | *P*-value |
|---|---|---|---|
| Age, years | 48.98±13.83 | 48.65±13.67 | 0.68 |
| Male, n(%) | 1432(60.12%) | 202(57.88%) | 0.43 |
| High school and above, n(%) | 1309(55.47%) | 200(57.31%) | 0.52 |
| Current and ever smoking, n(%) | 902(39.08%) | 141(41.59%) | 0.38 |
| Body mass index, kg/m2 | 24.51±3.63 | 24.39±3.51 | 0.58 |
| Systolic blood pressure, mmHg | 129.84±18.17 | 127.79±16.49 | 0.07 |
| Diastolic blood pressure, mmHg | 81.01±10.95 | 80.68±10.96 | 0.64 |
| Using anti-hypertensive medication, n(%) | 1425(73.42%) | 195(70.14%) | 0.25 |
| Diabetes mellitus, n(%) | 543(25.35%) | 82(26.71%) | 0.61 |
| History of CVD, n(%) | 242(10.16%) | 35(10.03%) | 0.94 |
| Creatinine, μmol/L | 143.05(100, 207) | 139(100, 203) | 0.60 |
| eGFR, ml/min/1.73m2 | 51.49±30.46 | 51.9±30.33 | 0.82 |
| eGFR<60ml/min/1.73m2, n(%) | 1620(68.01%) | 238(68.19%) | 0.94 |
| ACR, mg/g | 428.125(111.4, 982.15) | 511.71(131.7, 1058.9) | 0.17 |
| Albuminuria groups, n(%) | | | **0.04** |
| <30 mg/g | 287(12.32%) | 28(8.28%) | |
| 30-299 mg/g | 658(28.24%) | 112(33.14%) | |
| ≥300 mg/g | 1385(59.44%) | 198(58.58%) | |
| 24h urinary sodium excretion (mmol/24h) | 145.94±80.44 | 147.97±81.05 | 0.67 |
| Uromodulin, ng/mL | 91.03±61.31 | 95.52±61.7 | 0.23 |
| Etiology of CKD | | | 0.44 |
| Diabetic nephropathy | 345(14.90%) | 47(13.91%) | |
| Glomerulonephritis | 1390(60.02%) | 215(63.61%) | |
| Others | 581(25.09%) | 76(22.49%) | |

Note: Number of missing: education-22, smoking status-84, body mass index-263, SBP-379, DBP-379, using anti-hypertensive medication-512, diabetes mellitus-282, etiology of CKD-77, ACR-63, uromodulin-327.

Abbreviation: CVD, cardiovascular disease; eGFR, estimated glomerular filtration rate; ACR, albumin creatinine ratio ; CKD, chronic kidney disease.

**Table S4.** Characteristics of study participants between included and excluded participants

| Characteristics | Included participants n=2731 | Excluded participants* n=1146 | *P*-value |
|---|---|---|---|
| Age, years | 48.94±13.81 | 51.16±14.71 | **<0.001** |
| Male, n(%) | 1634(59.83%) | 666(58.12%) | 0.32 |
| High school and above, n(%) | 1509(55.70%) | 481(55.74%) | 0.99 |
| Current and ever smoking, n(%) | 1043(39.40%) | 271(32.81%) | **<0.001** |
| Body mass index, kg/m$^2$ | 24.50±3.62 | 24.60±3.68 | 0.51 |
| Systolic blood pressure, mmHg | 129.58±17.98 | 132.62±17.52 | **<0.001** |
| Diastolic blood pressure, mmHg | 80.97±10.95 | 80.59±11.09 | 0.37 |
| Using anti-hypertensive medication, n(%) | 1620(73.01%) | 460(70.77%) | 0.26 |
| Diabetes mellitus, n(%) | 625(25.52%) | 226(24.51%) | 0.55 |
| History of CVD, n(%) | 277(10.14%) | 76(6.63%) | **0.001** |
| Creatinine, μmol/L | 143(100, 207) | 146(101.5, 211.5) | 0.21 |
| eGFR, ml/min/1.73m$^2$ | 51.54±30.44 | 50.45±33.44 | 0.32 |
| eGFR<60ml/min/1.73m$^2$, n(%) | 1858(68.03%) | 815(71.12%) | 0.06 |
| ACR, mg/g | 435.59(114.00, 991.20) | 167.29(34.99, 556.04) | **<0.001** |
| Albuminuria groups, n(%) | | | **<0.001** |
| <30 mg/g | 315(11.81%) | 133(23.05%) | |
| 30-299 mg/g | 770(28.86%) | 210(36.40%) | |
| ≥300 mg/g | 1583(59.33%) | 234(40.55%) | |
| Uromodulin, ng/mL | 91.60±61.37 | 78.44±58.45 | 0.06 |
| Etiology of CKD | | | **0.005** |
| Diabetic nephropathy | 392(14.77%) | 116(15.83%) | |
| Glomerulonephritis | 1605(60.47%) | 478(65.21%) | |
| Others | 657(24.76%) | 139(18.96%) | |

* Note: Number of missing in the excluded population: education-283, smoking status-320, BMI-510, SBP-189, DBP-189, using anti-hypertensive medication-496, diabetes mellitus-224, etiology of CKD-413, ACR-569, uromodulin-1066.

Abbreviation: CVD, cardiovascular disease; eGFR, estimated glomerular filtration rate; ACR, albumin creatinine ratio ; CKD, chronic kidney disease.

**Table S5.** Genetic association between genotypes of rs4293393 or rs6497476 and eGFR or uromodulin

| Genetic variant | β (95%CI) for eGFR | β (95%CI) for uromodulin* |
|---|---|---|
| rs4293393 (GG vs. AA&GA) | -7.30(-19.06, 6.61) | -22.90(-45.93, 4.41) |
| rs6497476 (CC vs. TT&TC) | -2.21(-16.87, 13.94) | -15.68(-43.29, 18.03) |

Note: The results listed were gained by using bootstrap method after 500 times of sampling with replacement.
Abbreviation: eGFR, estimated glomerular filtration rate.
* There are 379 missing values for uromodulin.

**Table S6.** Genetic association between genotypes of rs11864909 or haplotypes composed of rs11864909, rs4293393 and rs6497476 and eGFR or uromodulin, by age, hypertension status, eGFR, ACR and etiology of CKD*

| Association model | β(95%CI) for eGFR | β(95%CI) for uromodulin** |
|---|---|---|
| rs11864909 (TT&TC vs. CC) | | |
| Age group | | |
| ≥65 years | 4.07(0.39, 7.65) | -11.82(-22.29, -1.92) |
| <65 years | 6.33(4.19, 8.36) | -19.78(-24.19, -15.79) |
| Hypertension | | |
| Yes | 5.09(2.66, 7.15) | -17.77(-22.33, -13.32) |
| No | 7.64(3.43, 11.53) | -19.93(-29.38, -10.88) |
| eGFR category | | |
| ≥60ml/min/1.73m$^2$ | 3.38(0.47, 5.93) | -28.14(-37.57, -19.12) |
| <60ml/min/1.73m$^2$ | 1.89(0.74, 3.03) | -12.87(-16.28, -9.6) |
| ACR category | | |
| <300mg/g | 3.20(0.54, 6.06) | -15.82(-23.26, -9.37) |
| ≥300mg/g | 7.21(4.53, 9.45) | -19.51(-24.29, -14.3) |
| Etiology of CKD | | |
| Diabetic nephropathy | 4.30(0.30, 8.20) | -13.99(-24.96, -4.82) |
| Glomerulonephritis | 7.09(4.40, 9.53) | -22.33(-27.42, -17.04) |
| Others | 1.48(-1.22, 4.10) | -12.73(-19.13, -6.66) |
| Haplotype composed of rs11864909, rs4293393 and rs6497476 (T-A-T vs. other types) | | |
| Age group | | |
| ≥65 years | 6.41(0.75, 11.87) | -17.23(-34.31, -0.63) |
| <65 years | 12.73(8.43, 16.43) | -36.9(-44.71, -28.99) |
| Hypertension | | |
| Yes | 8.51(4.67, 12.39) | -31.03(-39.69, -22.94) |
| No | 17.46(10.32, 24.23) | -38.75(-56.94, -22.88) |
| eGFR category | | |
| ≥60ml/min/1.73m$^2$ | 7.64(2.53, 11.92) | -50.16(-64.9, -35.11) |
| <60ml/min/1.73m$^2$ | 3.04(1.13, 5.13) | -21.89(-28.5, -16.4) |
| ACR category | | |
| <300mg/g | 6.13(1.71, 11.01) | -27.59(-40.17, -16.72) |
| ≥300mg/g | 13.91(9.42, 18.32) | -35.25(-44.3, -25.53) |
| Etiology of CKD | | |
| Diabetic nephropathy | 8.84(1.35, 16.15) | -28.97(-45.56, -13.5) |
| Glomerulonephritis | 13.57(8.66, 18.69) | -39.74(-49.66, -29.9) |
| Others | 2.48(-1.95, 6.48) | -21.04(-31.26, -10.91) |

Note: *The results listed were gained by using bootstrap method after 500 times of sampling with replacement. The association was adjusted for age, gender, smoking, body mass index, systolic blood pressure, using anti-hypertensive medication, diabetes mellitus, etiology of CKD, logarithm transformed urinary albumin

creatinine ratio, uromodulin and eGFR, as appropriate, when the variable was not used as dependent variable or for stratification.

Abbreviation: eGFR, estimated glomerular filtration rate; ACR, albumin creatinine ratio; CKD, chronic kidney disease.

** There are 379 missing values for uromodulin.

**Table S7.** Incidence rates for all-cause mortality, by genotypes of rs4293393 and rs6497476 and 24h urinary sodium excretion

| Genetic variant | 24h urinary sodium excretion<135mmol/24h | | | 24h urinary sodium excretion≥135mmol/24h | | |
|---|---|---|---|---|---|---|
| | No. of Events (%) | Rate/100 patient-years | *P*-value for log-rank test | No. of Events (%) | Rate/100 patient-years | *P*-value for log-rank test |
| rs4293393 | | | 0.50 | | | **0.005** |
| GA&GG | 7(3.78%) | 0.78 | | 1(0.52%) | 0.10 | |
| AA | 55(4.86%) | 1.01 | | 57(5.12%) | 1.02 | |
| rs6497476 | | | 0.63 | | | **0.007** |
| CT&CC | 6(3.92%) | 0.82 | | 1(0.56%) | 0.11 | |
| TT | 56(4.82%) | 1.00 | | 57(5.06%) | 1.01 | |

**Table S8.** eGFR slope stratified by genotypes of rs11864909, rs4293393 and rs6497476 and haplotypes involving the variants

| Genetic variant | eGFR slope (ml/min/1.73m$^2$ per year) | *P*-value for interaction between genetic variant and time |
|---|---|---|
| rs11864909 | | 0.58 |
| TT&TC | -2.02 | |
| CC | -1.76 | |
| rs4293393 | | 0.59 |
| GA&GG | -1.56 | |
| AA | -1.88 | |
| rs6497476 | | 0.48 |
| CT&CC | -1.44 | |
| TT | -1.89 | |
| Haplotype | | 0.55 |
| TAT | -1.76 | |
| Others | -2.26 | |

Abbreviations: eGFR, estimated glomerular filtration rate.

**Table S9.** eGFR slope stratified by genotypes of rs11864909, rs4293393 and rs6497476 and haplotypes composed of the variants among the participants with ≥3 times of measures of eGFR

| Genetic variant | eGFR slope (ml/min/1.73m$^2$ per year) | *P*-value for interaction between genetic variant and time |
|---|---|---|
| rs11864909 | | 0.58 |
| TT&TC | -1.92 | |
| CC | -1.64 | |
| rs4293393 | | 0.52 |
| GA&GG | -1.38 | |
| AA | -1.79 | |
| rs6497476 | | 0.55 |
| CT&CC | -1.37 | |
| TT | -1.78 | |
| Haplotype | | 0.50 |
| TAT | -1.63 | |
| Others | -2.23 | |

Abbreviations: eGFR, estimated glomerular filtration rate.

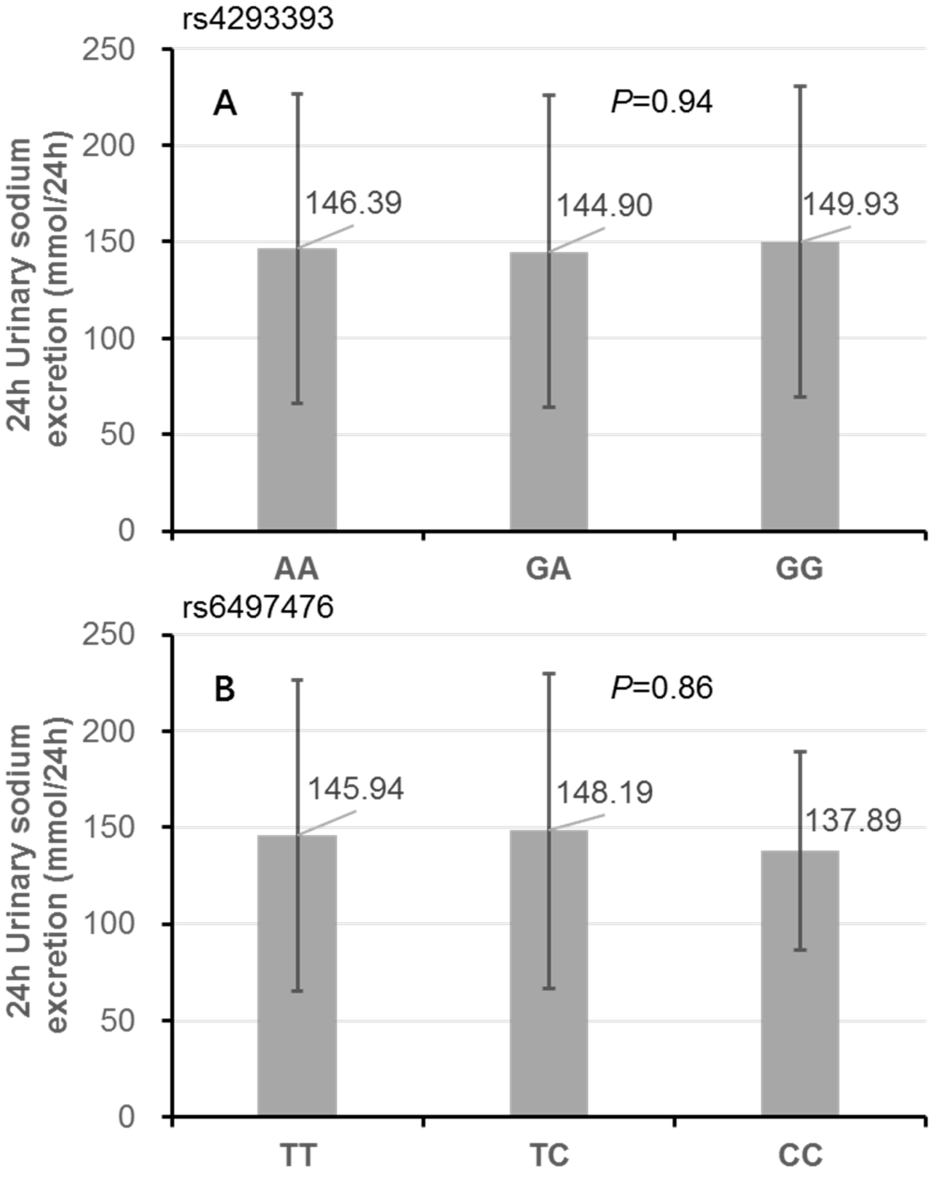


**Figure S1.** The distribution of 24-hour urinary sodium excretion through genotypes of rs4293393 and rs6497476. A. 24-hour urinary sodium excretion through genotypes of rs4293393; B. 24-hour urinary sodium excretion through genotypes of rs6497476.
